# Spatiotemporal Characteristics of 360-Degree Basic Attention

**DOI:** 10.1038/s41598-019-52313-3

**Published:** 2019-11-06

**Authors:** Yuki Harada, Junji Ohyama

**Affiliations:** Human Augmentation Research Center, National Institute of Advanced Industrial Science and Technology, Ibaraki, Japan

**Keywords:** Human behaviour, Health care

## Abstract

The spatiotemporal characteristics of basic attention are important for understanding attending behaviours in real-life situations, and they are useful for evaluating the accessibility of visual information. However, although people are encircled by their 360-degree surroundings in real life, no study has addressed the general characteristics of attention to 360-degree surroundings. Here, we conducted an experiment using virtual reality technology to examine the spatiotemporal characteristics of attention in a highly controlled basic visual context consisting of a 360-degree surrounding. We measured response times and gaze patterns during the 360-degree search task and examined the spatial distribution of attention and its temporal variations in a 360-degree environment based on the participants’ physical position. Data were collected from both younger adults and older adults to consider age-related differences. The results showed the fundamental spatiotemporal characteristics of 360-degree attention, which can be used as basic criteria to analyse the structure of exogenous effects on attention in complex 360-degree surroundings in real-life situations. For practical purposes, we created spherical criteria maps of 360-degree attention, which are useful for estimating attending behaviours to 360-degree environmental information or for evaluating visual information design in living environments, workspaces, or other real-life contexts.

## Introduction

The spatiotemporal characteristics of attention are important for understanding how people access visual information in real-life situations^1,2^. Knowledge of the spatiotemporal characteristics of attention will provide quantitative evaluation criteria for predicting how people pay attention to visual information and how their attention dynamically changes during visual search. From a practical perspective, the spatiotemporal characteristics of attention are useful for evaluating the accessible design of the visual information people are surrounded by, such as sign systems (e.g., the location of signs in facilities), interfaces (e.g., devices in the cockpits of vehicles), and content superimposed on real-life scenes (e.g., visual content for augmented reality glasses). To date, the spatiotemporal characteristics of attention have been investigated by laboratory and non-laboratory studies. However, both types of studies have potential limitations for fully understanding attentional characteristics.

Previous laboratory studies have investigated basic attention, which can be applied as a criterion for evaluating whether people can access information in specific contexts. For example, basic attention in a visual field is distributed in an elliptical manner around a fixation point (a radius of approximately 30–60 degrees)^3^, which is called the useful field of view (UFOV^4^). Because the size of the UFOV predicts the cause of daily accidents (e.g., vehicle crashes^5^), the UFOV is important from the perspective of accessible design^6–8^. However, the UFOV is limited with respect to fully predicting how people pay attention to visual information in real-life situations because it covers a radius of only 60 degrees around the fixation point^9^, while in real life, people are encircled by their 360-degree surroundings, including overhead, underneath, and behind. Moreover, there are many studies on spatiotemporal attention, but previous procedures have had difficulty expanding to 360-degree experiments for two main reasons. First, the target appearance area did not cover the entire 360-degree surrounding. Second, the display bezels or screen joints were visible on the target background, providing a visual context. For example, many previous studies have typically used a procedure in which a target stimulus was presented on a single display^9^ or multiple displays^10^, measuring the correct responses and response times (RTs) for the target and the gaze patterns. In such procedural conditions, participants can expect the possible locations of targets and choose to pay attention to those locations, and the bezels and joints between multiple displays become visual contexts that can influence attending behaviours. Therefore, to clarify the spatiotemporal characteristics of basic attention in real-life situations, it is necessary to construct new experiments to measure attending behaviours not only in the limited field of predictable visual conditions but also in the entire 360-degree field.

Previous non-laboratory studies have reported the spatiotemporal characteristics of 360-degree attention in specific contexts (driving a car^11^ and making tea^12^) by measuring gaze patterns while allowing participants to voluntarily pay attention to any direction; however, these studies are limited in explaining basic attention because the results were confounded by the environmental contexts. Indeed, attention is influenced by visual and auditory contexts, such as the presence of letters^13^ or pictures^14^; the spatial background^15^; memory of the context^16^; auditory signals^17^; and audio-visual cross-modality^18^. Therefore, to understand the spatiotemporal characteristics of 360-degree basic attention, it is necessary to measure the fundamental aspect of attention in a neutral context where the exogenous factors of specific contexts are reduced and highly controlled. The fundamental aspect of attention can become a criterion to extract, analyse, or evaluate the effects of the exogenous factors of complex specific contexts by comparing attending behaviours between basic characteristics in neutral contexts and specific characteristics in complex contexts. Therefore, to clarify the spatiotemporal characteristics of attention in specific contexts of real-life situations, it is important to measure the fundamental basic characteristics of how people pay attention to their 360-degree surroundings in simple neutral contexts.

To address these procedural limitations, we used a new experimental method that involved virtual reality technology to measure the spatiotemporal characteristics of 360-degree basic attention. Because virtual reality allows a target stimulus to be presented at any location in a seamless 360-degree environment, including above or underneath a participant, participants cannot expect a possible location of the target and cannot view any visual context. The present study fills the gap regarding the procedural limitations of laboratory and non-laboratory studies by applying virtual reality technology to investigate 360-degree attention in highly controlled basic visual contexts.

From a practical perspective, we applied a geometric analytical method of spherical complementation of discrete sampling data and a visualization technique to create 360-degree attention criteria maps. The maps are useful for designers and engineers to evaluate the design of visual information based on the spatiotemporal characteristics of basic attention. For example, augmented reality technology has been applied to support visual processing in various fields, such as driving^19^, surgery^20^, job training^21^, and education^22^. In these cases, the visual information of augmented reality should be located in quickly attended to areas in which the information can easily be acquired.

Attention can also be biased due to human factors such as sensory and cognitive ageing^23^ or disability (e.g., unilateral spatial neglect^24^). However, other previous studies imply that there are common characteristics of attention across different people. For example, the concept of the UFOV suggests that people’s general attention is most effectively distributed at the fovea and basically spreads horizontally and vertically, although the size of this distribution differs across people^9,13^. These reports on the characteristics of basic attention have been used as the valuation basis to understand differences in attentional bias related to the effects of ageing^3^ and disability^25^. In these studies, the basic attention of healthy young people was measured when environmental factors were controlled and was compared with attention in older individuals or people with a disability.

The purpose of this study was to provide knowledge of the spatiotemporal characteristics of 360-degree basic attention and a useful method for measuring 360-degree attention. In our experiment, the 360-degree attention of healthy younger adults (YAs) was measured in an environment where exogenous factors were reduced and highly controlled. We also measured the 360-degree attention of healthy older adults (OAs) in the same conditions to investigate whether the measured attentional characteristics were specific to YAs or common across ages. This study can be used as a reference and foundation for future studies on how 360-degree attention is influenced by exogenous and/or human factors and the mechanism of integrated complex conditions in real life.

**Figure 1:**
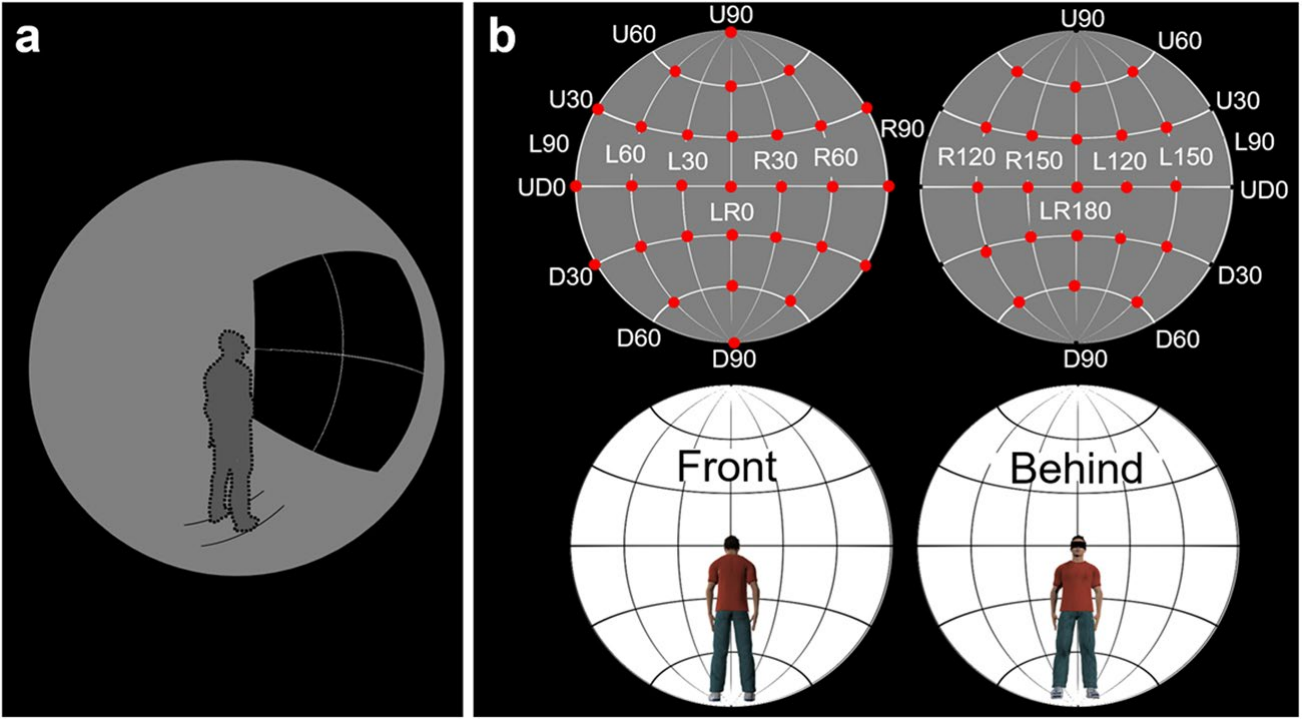
The experimental design in virtual reality. (**a**) Illustration of the ready phase. The dotted line represents a participant. (**b**) Target locations. The target was presented at one of the 50 red locations.

In our experiment, healthy YAs and OAs were asked to wear a head-mounted display (HMD) equipped with an eye-tracking system. After the participants pressed the start button in the ready phase (Fig. 1a), a target dot was presented at one of 50 locations (Fig. 1b). The participants’ task was to search for the target dot and to report the target colour by pressing a button as accurately and quickly as possible. We measured the correct responses and RTs for the targets to confirm the spatial characteristics of attention and the dynamic change in gaze patterns to confirm the temporal characteristics of attention. We analysed these data in two ways. First, to clarify the general spatiotemporal characteristics of basic attention in 360-degree surroundings, we analysed data that were merged across YAs and OAs. Second, to clarify the age-related differences in the spatiotemporal characteristics of basic attention, we analysed these data for each age group.

## Results

### General spatiotemporal characteristics of 360-degree attention

To clarify the general spatiotemporal characteristics of 360-degree basic attention regardless of age, we merged all of the data on correct responses, RTs, and gaze patterns across 15 YAs and 19 OAs. The mean correct response rate for all target locations was 0.98 (*SD* = 0.011), indicating that the participants accurately reported almost all of the targets (see Supplementary Table S1 for the correct response rate for each location).

#### RT data

**Figure 2:**
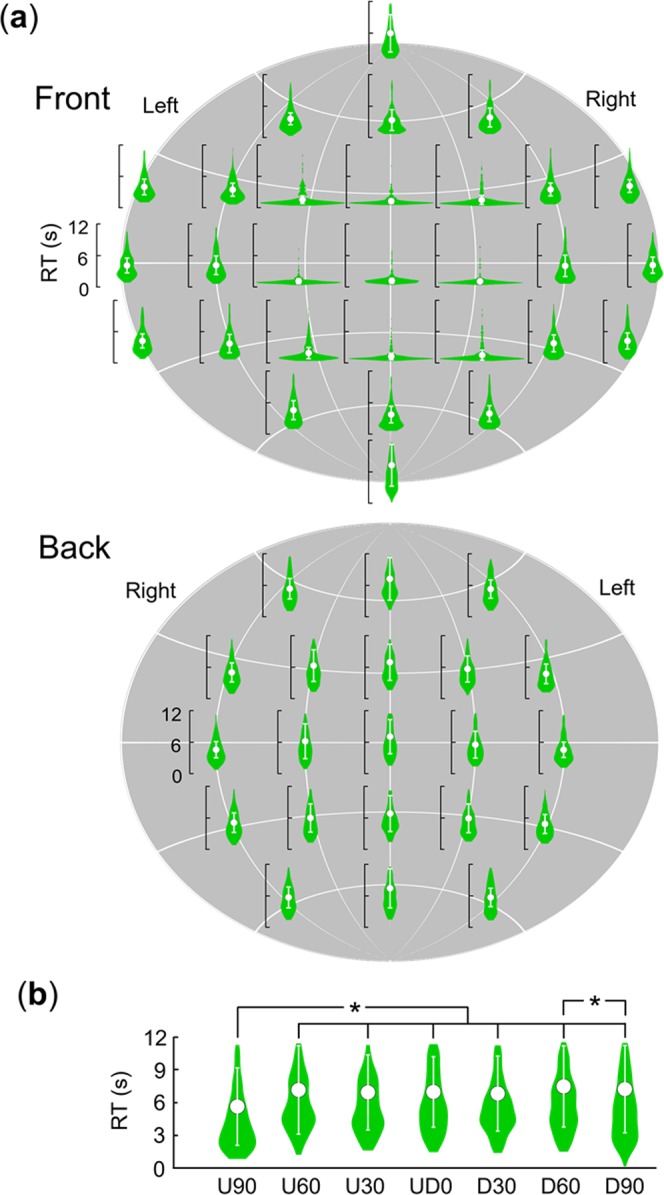
The means and standard deviations of the RTs in the 34 participants. (**a**) All target locations. (**b**) A line of the LR180. White circles represent the mean RTs, and the error bars represent the standard deviations. Violin plots represent the Kernel density estimation. Asterisks represent significant differences.

Figure 2 shows the mean RTs across the 34 participants for the 50 locations (see Supplementary Table S2 for the values). To clarify the general spatial aspects of 360-degree basic attention, we conducted a one-way within-participants analysis of variance (ANOVA) on log-transformed RTs with a fixed effect of target location (50) and with random effects of participant and colour. The results revealed that the main effect was significant (*F* (49, 13224) = 271.24, *p* < 0.0001). A multiple comparison revealed that, for horizontal lines U60, U30, UD0, D30, and D60, the RTs were significantly faster at a target location than at a neighbour location far from LR0 (*t*s (13224) > 2.17, *p*s < 0.030, *d*s > 0.05), except for L60 vs. L90 on UD0 (*t* (13224) = 1.72, *p* = 0.086, *d* = 0.02) and LR0 vs. L30 or R30 (faster at L30 or R30 than at LR0: *t*s (13224) > 3.64, *p*s < 0.0003, *d*s > 0.47). As with horizontal lines, on the vertical line of LR0, the RTs were significantly faster at a target location than at a neighbour location far from UD0 (*t*s (13224) > 2.10, *p*s < 0.036, *d*s > 0.22). The multiple comparison also revealed that, on the vertical line of LR180, the RTs were significantly faster at U90 than from U60 to D90 (*t*s (13224) > 6.08, *p*s < 0.0001, *d*s > 0.34) and significantly faster at D90 than at D60 (*t* (13224) = 2.42, *p* = 0.016, *d* = 0.07). The other results of multiple comparisons are shown in Supplementary Data 1.

**Figure 3:**
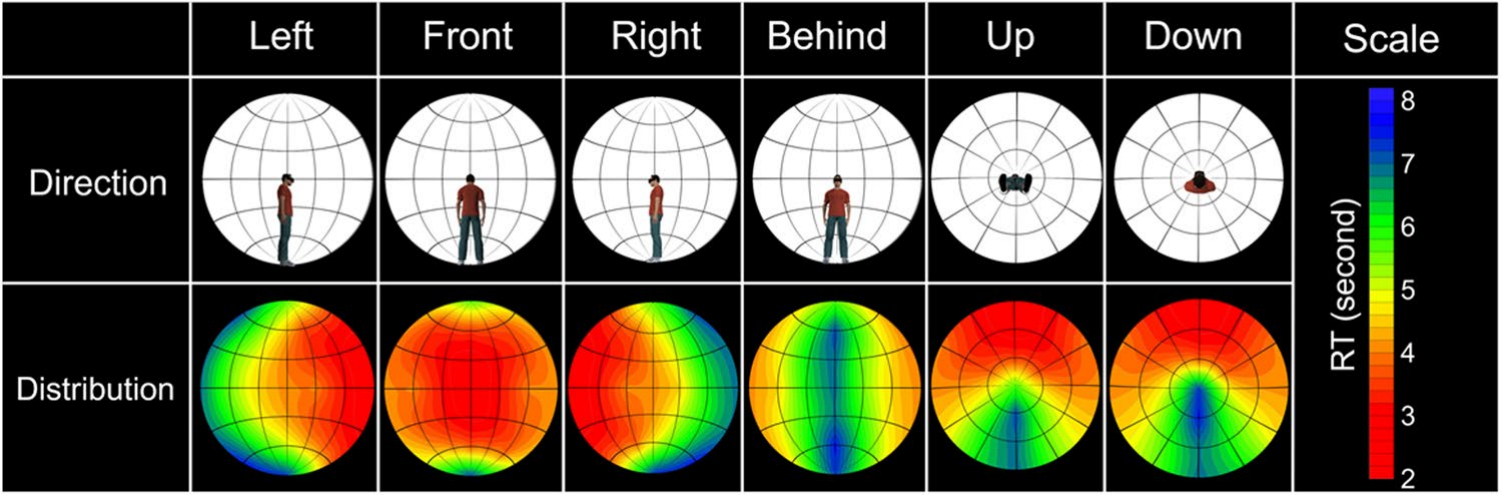
360-degree attention criteria heat maps based on the mean RTs merged across age groups. Each direction illustrates a viewing point from six different directions related to the participants’ physical position. Hemispherical heat maps show estimated distribution of the 360-degree attention complement from measured mean RTs.

Based on the RT data, we created 360-degree attention criteria maps (Fig. 3: see Creation of attention criteria maps for more details). These criteria maps support the results of the statistical analysis that spatial attention is most effectively distributed in the UFOV, spreads gradually in horizontal and vertical directions, and is least effectively distributed behind people.

#### Eye movement data

**Figure 4:**
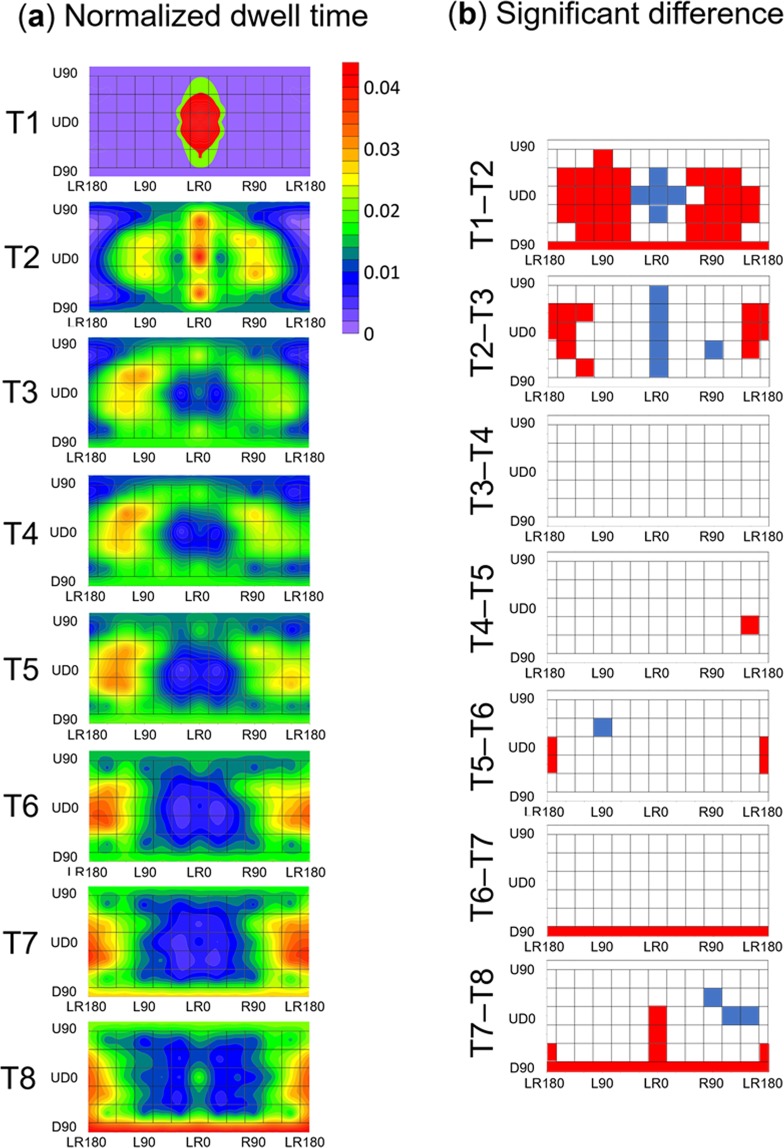
Temporal characteristics of 360-degree spatial attention merged across age groups. (**a**) Normalized dwell time for each time phase and area. The colour scale is identical across the time phases (T1–T8). (**b**) Significant difference in normalized dwell times between different time phases. Red areas represent significantly longer times spent dwelling on an area compared with that spent on the same area one time before (*p* < 0.05) while blue areas represent the opposite.

To clarify the temporal aspect of basic attention in 360-degree surroundings, we analysed the dynamic move of gaze patterns and its time sequence as follows. First, the time series of gaze patterns were divided into eight phases in the time course (i.e., T1–T8). Second, the entire 360-degree field was divided into 62 areas by spherical coordination grids (5 vertical levels × 12 horizontal levels + U90 and D90). Third, the normalized dwell times of the gaze of each participant were calculated in each of the eight temporal phases and each of the 62 spatial areas. Figure 4a shows heat maps of the normalized dwell times for each time phase and area. To investigate how 360-degree attention changed over time, a two-way within-participants ANOVA was conducted on the normalized dwell time with the fixed effects of area (62: U60, U30, UD0, D30, D60 × L150, L120, L90, L60, L30, LR0, R30, R60, R90, R120, R150, LR180 + U90 and D90) and time phase (8: T1–T8) and with the random effect of participant. The results showed that the main effect of area and the two-way interaction were significant (*F* (61, 2046) = 40.71, *p* = 0.0001 and *F* (7, 14322) = 58.48, *p* < 0.0001, respectively). The main effect of time phase was not significant (*F* (7, 14322) = 0.25, *p* = 0.97). A multiple comparison showed how 360-degree attention changed with time (Fig. 4b). Attention was mainly paid to the area near UD0 and LR0 at T1 and was moved horizontally to the back of participants from T2-T6. After the horizontal moving, attention was paid to the area underneath participants from T7-T8.

Figure 4a shows slightly redder colour gradations to the left than to the right, especially for T3-T5. On this point, previous studies have reported that healthy adults’ attention is biased leftward^26,27^. To examine this issue, a three-way within-participants ANOVA was conducted on the normalized dwell time with fixed effects of direction (left, right), area (25: U60, U30, UD0, D30, D60 × L30/R30, L60/R60, L90/R90, L120/R120, and L150/R150), and time phase (T1–T8) and with the random effect of participant. The results revealed that the main effects of area and time phase were significant (*F* (24, 813.2) = 29.80, *p* < 0.0001 and *F* (7, 239.7) = 146.31, *p* < 0.0001, respectively). The results also revealed that the two-way interactions were significant between area and direction (*F* (24, 12095.1) = 1.57, *p* = 0.038), between direction and time phase (*F* (7, 12095.1) = 11.87, *p* < 0.0001), and between area and time phase (*F* (168, 12095.1) = 16.55, *p* < 0.0001). For the effect of direction on 360-degree attention, a multiple comparison revealed that the normalized dwell time was significantly larger for the left side than for the right side at T3 (*t* (756) = 4.77, *p* < 0.0001, *d* = 0.70), T4 (*t* (756) = 4.68, *p* < 0.0001, *d* = 0.54), and T5 (*t* (756) = 3.83, *p* = 0.0002, *d* = 0.51). In contrast, the normalized dwell time was significantly larger for the right side than for the left side at T1 (*t* (756) = 2.70, *p* = 0.007, *d* = 0.36) and T7 (*t* (756) = 2.81, *p* = 0.005, *d* = 0.41). Neither the main effect of direction nor the three-way interaction was significant (*F*s < 3.10, *p*s > 0.084).

### Age-related differences in the spatiotemporal characteristics of 360-degree attention

#### RT data

**Figure 5:**
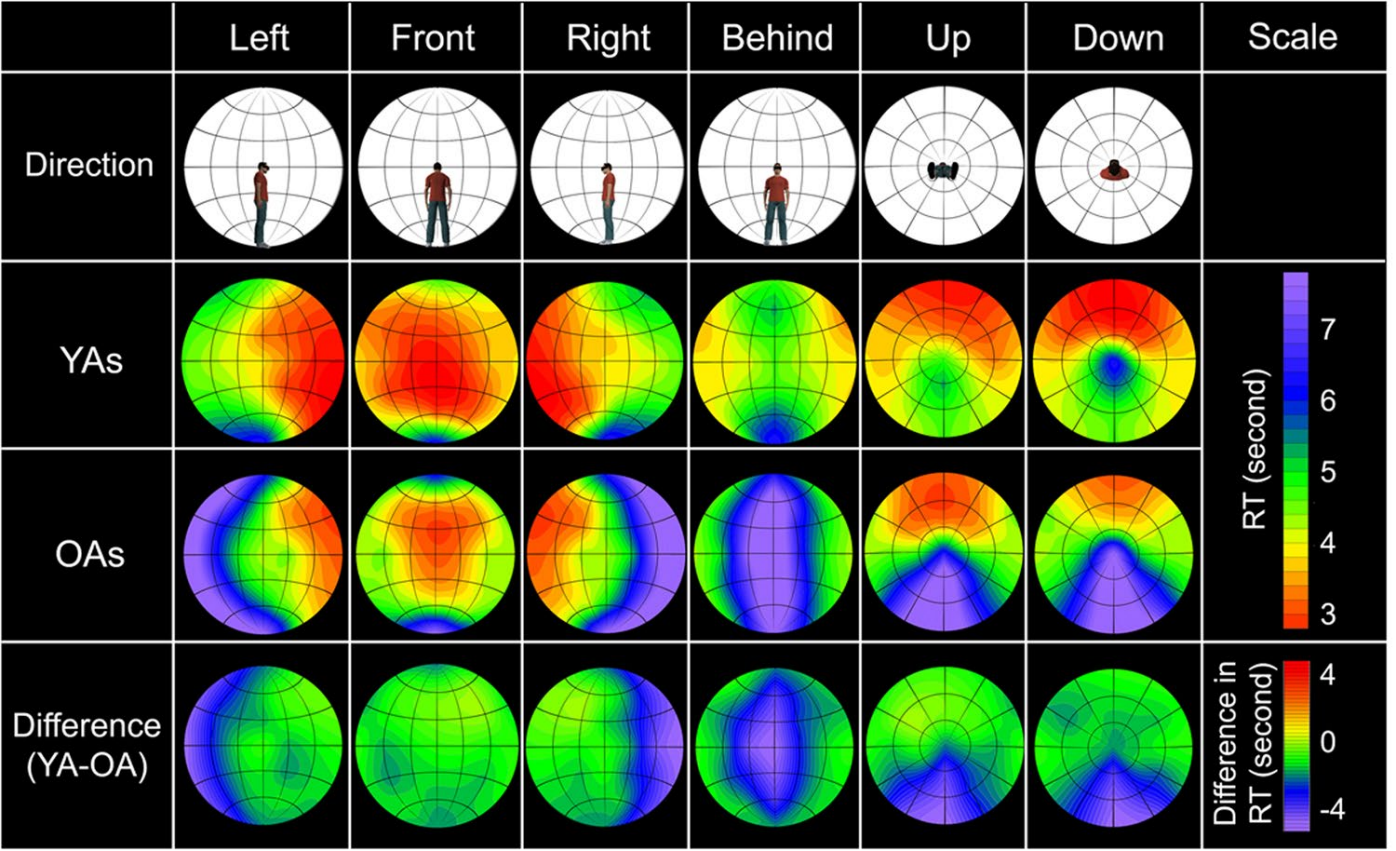
360-degree attention criteria maps based on the mean RTs in 15 YAs and 19 OAs. Heat maps of YAs and OAs show estimated distribution of the 360-degree attention complement from measured the mean RTs of each age group. Difference heat maps were created by subtracting the mean RTs of OAs from those of YAs.

**Figure 6:**
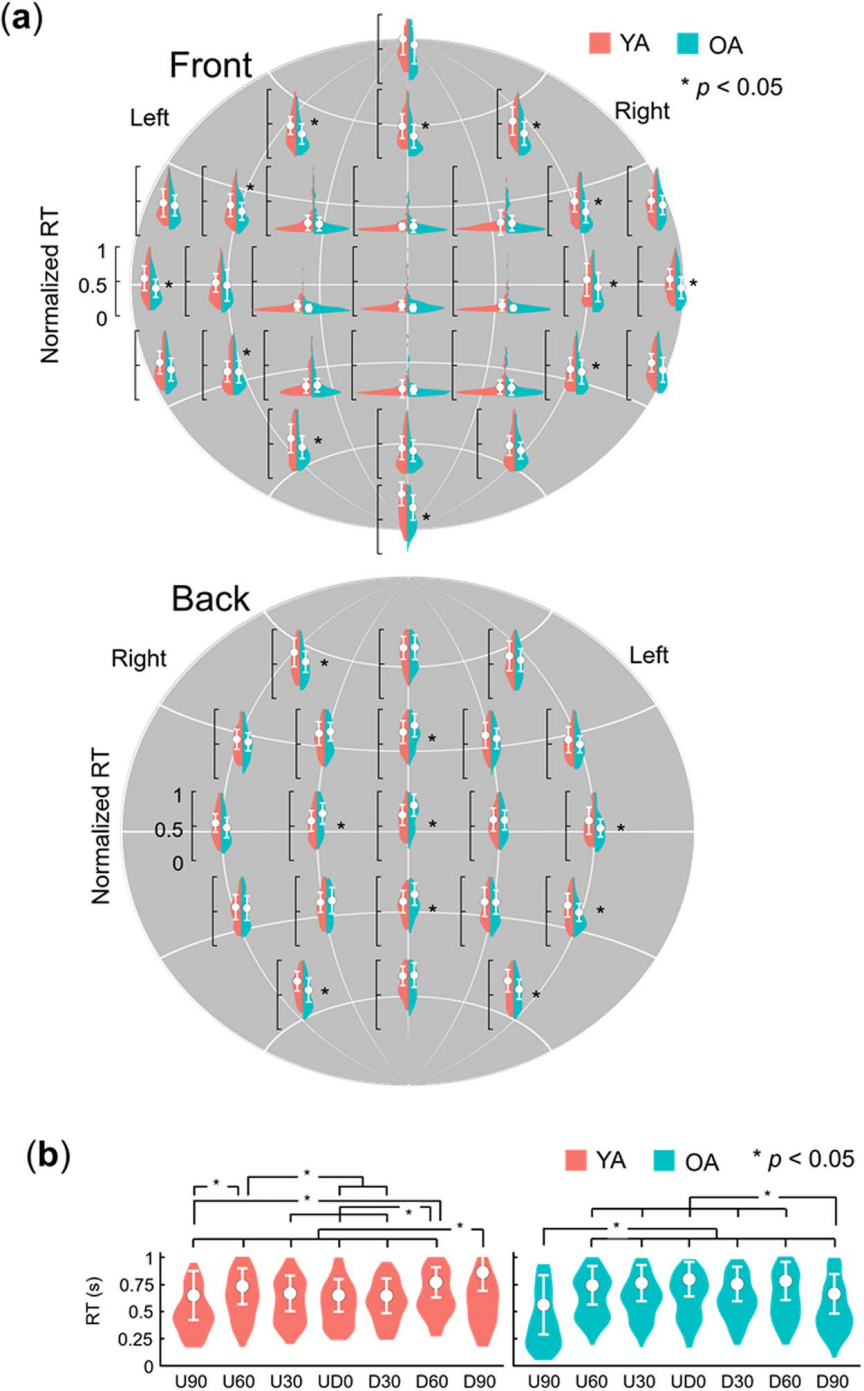
The means and standard deviations of the normalized RTs in 15 YAs and 19 OAs. (**a**) All target locations. White circles represent the mean RTs, and the error bars represent the standard deviations. Violin plots represent the Kernel density estimation. Asterisks represent significant differences between the YAs and OAs. (**b**) A line of LR180. Asterisks represent significant differences between different locations.

**Figure 7:**
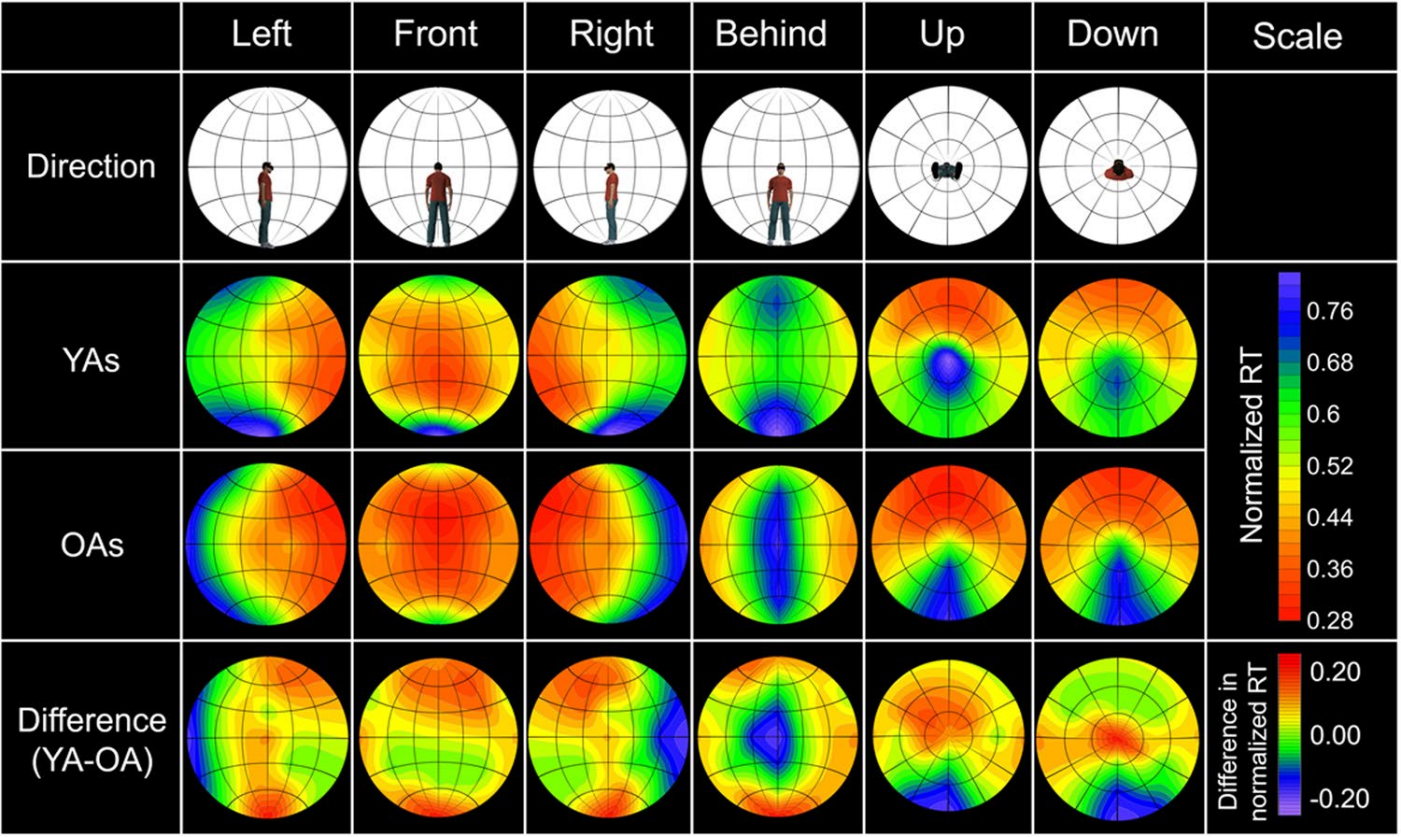
360-degree attention criteria maps based on the normalized RTs in 15 YAs and 19 OAs. Heat maps of YAs and OAs show estimated distribution of the 360-degree attention complement from measured the mean normalized RTs of each age group. Difference heat maps were created by subtracting the mean normalized RTs of OAs from those of YAs.

To clarify age-related differences in the spatial aspect of basic attention in 360-degree surroundings, we calculated the mean RTs at the 50 locations for each age group (Supplementary Fig. S1; see Supplementary Tables S5 and S6 for the values) and created 360-degree attention criteria maps by using the same procedure (Fig. 5). We also created difference heat maps by subtracting RTs of OAs from those of YAs. The maps show that OAs produced an entirely slower response than YAs, because of processing speed of visual stimuli^28^ or the requirement of quickly responding. To compare spatial attention between the age groups regardless of processing speed, we normalized the RTs (Fig. 6; see Supplementary Tables S7 and S8 for the values) by dividing the RTs for each location by the mean RT across repetitions of the slowest location. Based on the normalized RT data, we created 360-degree attention criteria maps (Fig. 7). We conducted a two-way mixed ANOVA on the normalized RTs with the fixed effects of age group (YAs, OAs: between participants) and target location (50) and with the random effects of participant and colour. The main effects of target location and age were significant (*F* (49, 13175) = 121.53, *p* < 0.0001 and *F* (1, 32) = 7.30 *p* = 0.012, respectively). The results also revealed that the two-way interaction was significant (*F* (49, 13175) = 5.28, *p* < 0.0001). A multiple comparison revealed that the normalized RTs were significantly larger for the OAs than the YAs at the 4 locations behind the participants (*t*s > 2.19, *p*s < 0.030, *d*s = 0.59) and were smaller for OAs than YAs at 18 locations (*t*s > 2.26, *p*s < 0.025, *d*s > 0.03). The other multiple comparison results are shown in Supplementary Data 2 (YAs) and 3 (OAs).

#### Eye movement data

**Figure 8:**
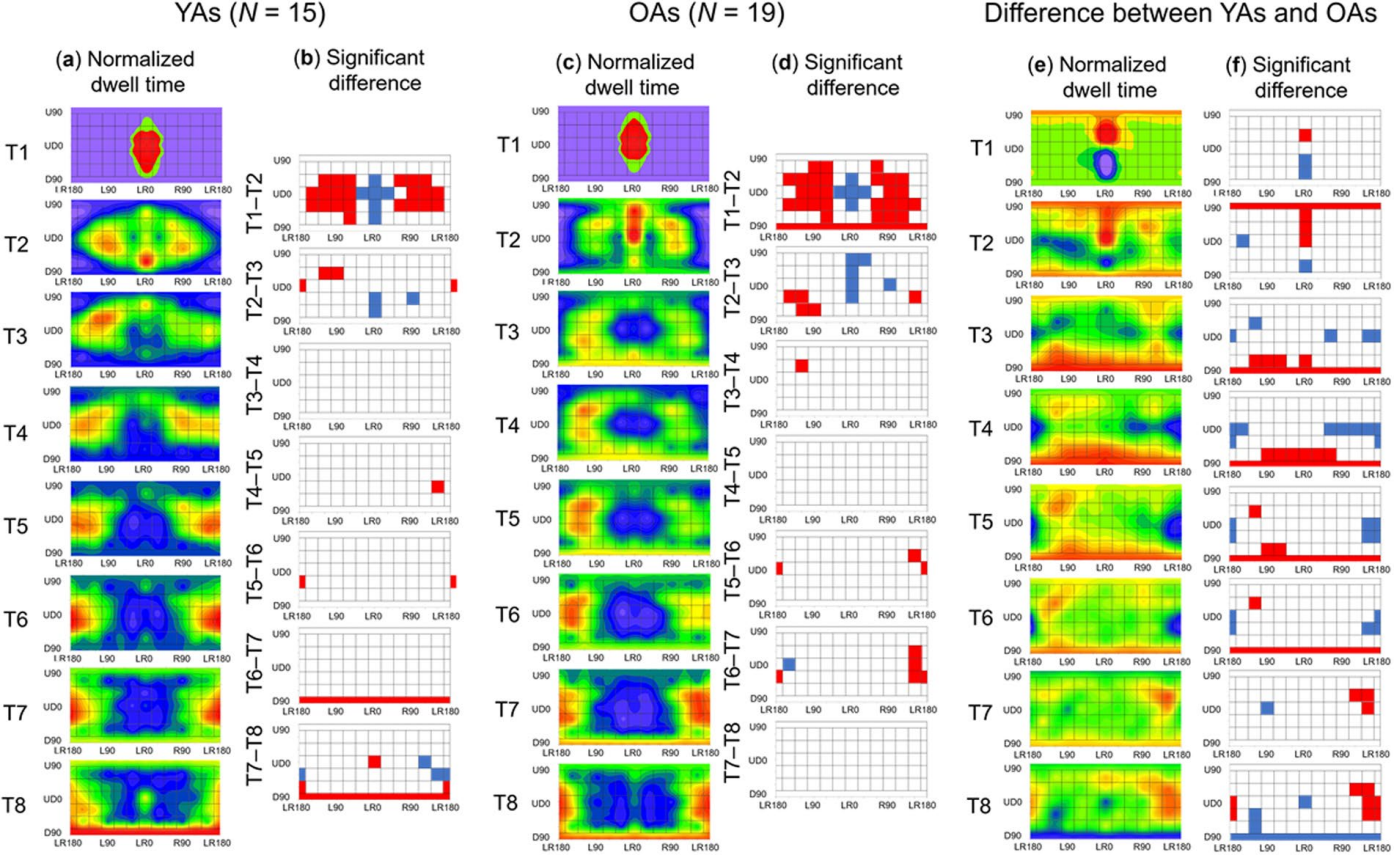
Temporal characteristics of 360-degree spatial attention in YAs and OAs. (**a**) The heat maps of the dwell time for the YAs. (**b**) Significant differences in normalized dwell time between different time phases in YAs. (**c**) The heat maps of the dwell time for the OAs. In (**a**,**c**), colour scales are the identical to Fig. 4a. (**d**) Significant differences in normalized dwell time between different time phases in OAs. In (**b,d**), the red areas represent a significant increase in dwell time (*p* < 0.05), and the blue areas represent a significant decrease in dwell time (*p* < 0.05). (**e**) The difference in h**e**at maps of dwell times between YAs and OAs. The range of colour scale is from −0.055 (the reddest) to 0.035 (the most violet). (**f**) Significant differences in normalized dwell times between YAs and OAs. The red areas show that the dwell time was significantly longer in the OAs than in the YAs (*p* < 0.05), and the blue areas show the opposite.

To clarify age-related differences in the temporal aspect of 360-degree basic attention, we created heat maps of the dwell time for each age group (Fig. 8a,c,e). According to Fig. 8a,c, the OAs attended to the area beneath them (D60, D90) before attending to the area behind them (LR180), but the YAs did the opposite. We conducted a three-way mixed ANOVA on the normalized dwell time with the fixed effects of age group (YAs, OAs: between participants), area (62), and time phase (T1–T8) and with the random effect of participant. The results revealed that the main effect of area was significant (*F* (61, 1984) = 40.92, *p* < 0.0001). The two-way interactions were significant between age and area (*F* (61, 1952) = 1.55, *p* = 0.004) and between area and time phase (*F* (427, 13888) = 58.41, *p* < 0.0001), and the three-way interaction was significant (*F* (427, 13888) = 1.42, *p* < 0.0001). The main multiple comparison results are shown in Fig. 8b,d,f. Neither the main effect of time phase nor the other interaction was significant (*F*s (13888) < 0.20, *p*s > 0.99).

## Discussion

In the present study, we investigated the spatiotemporal characteristics of basic attention in a 360-degree environment in which exogenous factors were reduced and highly controlled. Major findings of general characteristics across age groups are that (a) 360-degree spatial attention is most effectively distributed in the UFOV, spread gradually in horizontal and vertical directions, and least effectively distributed behind people and that (b) the spatial attention is dynamically moved from a front of people to the back and underneath them. Additionally, a major finding of age-related differences in the spatiotemporal characteristics is that (c) attention of OAs is moved to beneath them before to behind them, but that of YAs is the opposite.

RT data suggest that spatial attention is most effectively distributed in the UFOV (i.e., between U30/L30 and D30/R30) and spread gradually in horizontal and vertical directions. These results are consistent with eye movement data, showing that spatial attention was moved horizontally and vertically with time. RT and eye movement data also show that attention is less distributed behind people in the early time phase. These spatiotemporal characteristics of attention can be interpreted as an adaptive function^29^ for surviving in the primitive world. It is reasonable for people to pay attention to that which is ahead of them to avoid confronting a possible threat before approaching closer to it rather than attending to that which is slightly behind them.

Eye movement data show the left-attending bias in 360-degree fields when participants can voluntarily attend to any location. This left-attending bias may be related to the superiority of the right hemisphere, which specializes in visuospatial attention to the left^24^. For example, healthy people have a tendency to estimate the midpoint to the left of the horizontal centre (pseudo-neglect^26,27^). The superiority of the right hemisphere is influenced by handedness: attention is biased leftward in right-handed individuals but not in left-handed individuals^30^. Consistent with the handedness explanation, the participants showed left-attending bias because most of them were right-handed (*N* = 30). We did not conduct further examinations of the handedness explanation because doing so falls beyond the scope of our study; however, further studies will be required to clarify the relationship between handedness and 360-degree basic attention.

The main results confirm that the spatiotemporal characteristics of 360-degree attention are general across ages. However, our results also suggest age-related differences in the spatiotemporal characteristics of 360-degree attention. The comparison of normalized RTs between the age groups show that OAs produced slow responses for targets presented to the area behind them (Fig. 6). Eye movement data show that OAs’ attention focused on the area above LR0 from T1–T2, moved to the area underneath them from T3–T6, and was finally moved to the area behind them (Fig. 8f). In contrast, YAs’ attention was paid to the area below LR0 from T1–T2, horizontally moved to the area behind them from T3–T6, and was finally moved to the area underneath them. These results imply that OAs’ attention is less distributed to the area behind them in the early time phase. This distribution of attention may be related to the idea that it is difficult for OAs to disengage their attention from an area and shift it to another area^23,31^. Accordingly, OAs delay disengaging their attention when it is directed toward the frontal area of their 360-degree surroundings and moving it to the area behind them, resulting in the preferred sequence of attendance.

The findings are consistent with previous reports of the limited spatial characteristics of the basic UFOV, such as findings that the shape of the UFOV was generally similar between YAs and OAs, but the size was smaller in OAs^3^. Because the purpose of the present study was to provide knowledge of basic 360-degree attention based on logic similar to that applied in basic studies of the UFOV, our study cannot explain the effects of comprehensive factors across the wide range of ages (e.g., educational experience of the elderly, child development). Further studies are required to clarify the comprehensive factors, but our present findings and experimental method could be used as an initial standard for the study of the effects of environmental and human factors on 360-degree attention.

Based on the results, we created 360-degree attention criteria maps that illustrate the spatiotemporal characteristics of 360-degree basic attention as heat maps. The 360-degree attention criteria maps provide a standard for estimating how people pay attention in 360-degree fields. For example, augmented and virtual reality technologies have been applied to various fields. In these cases, the augmented and virtual information should be located in the redder areas in which the information can quickly be acquired. From a practical perspective, the maps can be useful for designers and engineers to evaluate the design of visual information with various types of content to enhance its accessibility.

## Methods

### Participants

Sixteen healthy Japanese YAs (14 males and 2 females; 15 right-handed and 1 left-handed) aged 18–25 years (*M* = 22.50, *SD* = 2.00) and nineteen healthy Japanese OAs (10 males and 9 females; 16 right-handed and 3 left-handed) aged 62–69 years (*M* = 67.05, *SD* = 2.46) were given a reward for their participation. All participants reported that they had normal or corrected-to-normal vision (range: 0.5–1.5 degrees, as measured by a Landolt test), and they were naïve to the purpose of our experiment. The experiment was approved by the safety management department of the National Institute of Advanced Industrial Science and Technology and conducted according to the principles of the Declaration of Helsinki. Before the experiment, written consent was obtained from all participants. The sample size of each group was determined based on previous studies investigating attention (*N*s = 20, 17, and 14 in three experiments^32^; *N*s = 10 and 12 in two experiments^33^). A post hoc power test was performed with G power and showed that the power was 1.00. We set the parameters as follows: statistical test = “ANOVA: Repeated measures, within-between interaction”, effect size = 0.25, alpha = 0.05, total sample size = 34, number of groups = 2, and number of measurements = 50.

### Apparatus and stimuli

The stimuli were displayed on an HMD (based on HTC Vive, SensoMotoric Instruments) that was equipped with an eye-tracking system and controlled by Experiment Center 4.0 (SensoMotoric Instruments), which was installed on a desktop PC (Alienware Area-51, DELL). The participants gave their responses on two wireless game pads (Dualshock 4, Sony Interactive Entertainment).

To measure the spatiotemporal characteristics of 360-degree basic attention, we used a 360-degree search task. The target stimuli were white ([R, G, B] = [255, 255, 255]) and black ([R, G, B] = [0, 0, 0]) dots (1.5 × 1.5 visual angle degrees) that were superimposed on a grey background ([R, G, B] = [128, 128, 128]). In this study, we did not use shaped targets^34,35^ as target stimuli because the orientation of a shaped target would be changed by the participants’ posture. The target locations were defined by a combination of vertical and horizontal levels (Fig. 1b), each of which was specified as an angle. There were seven target dots at the vertical level (up: U90, U60, and U30; Down: D30, D60, and D90; and UD0 angles), twelve target dots at the horizontal level at U30, UD0, and D30 (left: L150, L120, L90, L60, and L30; right: R30, R60, R90, R120, and R150; and LR0 and LR180 angles), six at U60 and D60, and one at U90 and D90. In total, there were 50 target locations.

In the ready phase (Fig. 1a), the participants were presented with a white cross embedded in a black square (90 × 90 visual angle degrees) with two parallel black lines (30 visual angle degrees in both lengths) on the floor to guide the initial foot positioning (10 visual angle degrees between the two black lines), followed by a black fixation cross (1.5 × 1.5 visual angle degrees).

### Procedure

This experiment was conducted in a room at the National Institute of Advanced Industrial Science and Technology. After receiving the experimental instructions and signing an informed consent form, the participants were asked to wear the HMD and to hold the game pad in each hand. The experiment was performed within one day (approximately three hours) for YAs or two days (approximately six hours) for OAs.

The sequence of an experimental trial was as follows. In the ready phase, the participants turned their body to the centre of the white cross embedded in a black square. After pressing the start button, the ready phase continued for 1000 ms. Then, a fixation cross was presented for 500 ms, and a white or black target dot was presented at one of the 50 possible locations. The participants’ task was to search for the target dot and to report the target colour by pressing a button (“L1” or “R1”) as accurately and quickly as possible. The requirement of responding quickly was based on previous studies^29,36^. The combination of the target button and the dot colour was counterbalanced across participants. In the target phase, the participants were allowed to move their body to search for the target. The RTs represent the spatiotemporal characteristics of attention because the RTs become longer as participants spend more time searching for a target. After a target button was pressed, the target disappeared, and the next trial began.

There were 400 trials: 50 target locations × 2 target colours × 4 experimental blocks. Each dot location was repeated 8 times (twice per block × 4 blocks) for each participant. The order of the trials was randomized across blocks and participants. Due to fatigue, OAs conducted two blocks per day, while YAs conducted four blocks per day. The participants rested for an interval between blocks and were allowed to pause any time during the experimental sessions.

Although the 360-degree visual search task cannot exclude a small perceptual effect of target colour discrimination, the results of our study reflect the characteristics of attention well and are relatively unaffected by perceptual effects for three reasons. First, target colour discrimination has frequently been used to measure attention, for example, in visual search tasks^2^. Second, the gaze patterns during the target search represent the characteristics of attention even though the task is colour discrimination. Third, we obtained results indicating that the target colour did not interact with either target location or age on RTs. A three-way mixed ANOVA was conducted on the normalized RTs with fixed effects of age group, target colour, and location and with a random effect of participant. Neither the main effect of colour nor any interaction between colour and the other factors was significant (*F*s < 3.16, *p*s > 0.075).

### Data exclusion

All the data obtained from one younger male were excluded because he did not fixate on the fixation cross due to a misunderstanding with respect to the experimental instructions. For each participant, we excluded the RTs obtained from incorrect responses and extremely slow RTs (approximately the slowest 1% of the RTs), as in previous studies^17,36,37^, because they were considered outliers. For 11 YAs, we excluded gaze data obtained from trials in which the target dot was black and located at U30 × R150 because we could not record these them due to program errors.

### Statistical analysis

The software R was used for statistical tests. ANOVAs were conducted with the generalized linear mixed model using the “lmer” function in the “lmerTest” library. Multiple comparisons were conducted using the “difflsmeans” function in the “lsmeans” library, and *p* values were adjusted with Holm’s method.

### Creation of attention criteria maps

To create the heat maps of attention criteria and the dwell time, Surfer 15.3 (Golden Software) was used to interpolate the contour lines. The maps are considered to represent the spatiotemporal characteristics of 360-degree basic attention because the interpolation was based on RTs obtained from observed locations.

To create the attention criteria heat maps, we linearly interpolated twelve missing RTs at L150, L90, L30, R30, R90, and R150 along the line defined by U60 and D60. Then, we removed the RTs at nine locations (U30, UD0, and D30 in L30, LR0, and R30) and interpolated them using data outside the nine locations to eliminate the effect of the UFOV, because numerous studies have examined the spatial aspect of attention in the UFOV. In the present study, the size of the UFOV was operationally defined as the area around the fovea, for which the vertical and horizontal angles were less than 60 degrees, based on previous studies^3,9^. The RTs were converted into grid data (horizontal: 360 degrees, vertical: 90 degrees) using a linear variogram model (the default setting of Surfer). The contour lines of the map were estimated by interpolating the points with equal RTs. To illustrate the attention criteria map, the “rainbow colour”, “reverse”, and “logarithmic scaling” settings were used in Surfer. The attention criteria map was converted into a 3D spherical image using a 3D viewer (THETA, RICOH).

## Supplementary information

Supplementary Info

Dataset 1

Dataset 2

Dataset 3

## Data Availability

Additional data related to this paper may be requested from the corresponding author.
