# Supplementary material for: Spatiotemporal Characteristics of 360-Degree Basic Attention
Source: Sci Rep. 2019 Nov 6;9:16083. doi: 10.1038/s41598-019-52313-3 (PMC6834598; doi:10.1038/s41598-019-52313-3)
Supplement: Supplementary file 1 — Supplementary Info [file 41598_2019_52313_MOESM1_ESM.docx]

Spatiotemporal Characteristics of 360-degree Basic Attention

**Supplementary Information**

Yuki, Harada & Junji, Ohyama

Human Augmentation Research Center, National Institute of Advanced Industrial Science and Technology, Ibaraki, Japan

*Corresponding author: [j.ohyama@aist.go.jp](mailto:j.ohyama@aist.go.jp)

**Supplementary Data 1. The statistical values of a multiple comparison between 50 locations for the merged RTs.** A one-way ANOVA was conducted on the log-transformed RTs merged across 15 YAs and 19 OAs with the factor target location (50). The results revealed that the main effect was significant. Supplementary Data 1 shows the statistical values of a multiple comparison for the main effect.

**Supplementary Data 2. The statistical values of a multiple comparison between 50 locations for the normalized RTs of the YAs.** A two-way ANOVA was conducted on the normalized RTs with the factors age group (YAs, OAs) and target location (50). The results revealed that the two-way interaction was significant. Supplementary Data 2 shows the statistical values of a multiple comparison for the simple main effect of target location on YAs.

**Supplementary Data 3. The statistical values of a multiple comparison between 50 locations for the normalized RTs of the OAs.** As in Supplementary Data 2, Supplementary Data 3 shows the statistical values of a multiple comparison for the simple main effect of target location on OAs.

**Supplementary Table**

Table S1. The mean correct response rate of the 360-degree search task merged across 15 YAs and 19 OAs.

|  | Longitude | | | | | | | | | | | | |
| --- | --- | --- | --- | --- | --- | --- | --- | --- | --- | --- | --- | --- | --- |
|  |  | L150 | L120 | L90 | L60 | L30 | LR0 | R30 | R60 | R90 | R120 | R150 | LR180 |
| Latitude | U90 |  |  |  |  |  | .96 (.09) |  |  |  |  |  |  |
|  | U60 |  | .99 (.05) |  | .99 (.04) |  | .99 (.04) |  | 1.00 (.00) |  | .98 (.04) |  | .99 (.04) |
|  | U30 | .97 (.07) | .99 (.04) | .97 (.05) | .98 (.06) | .99 (.07) | .99 (.04) | 1.00 (.02) | .98 (.07) | 1.00 (.02) | .99 (.04) | .97 (.08) | .98 (.06) |
|  | UD0 | .98 (.04) | .99 (.04) | .99 (.05) | .99 (.04) | .97 (.06) | .98 (.05) | .98 (.05) | .99 (.04) | .99 (.04) | .98 (.04) | .98 (.05) | .99 (.04) |
|  | D30 | .97 (.06) | .98 (.05) | .99 (.04) | .98 (.05) | .99 (.04) | .99 (.03) | .98 (.04) | .98 (.04) | .99 (.04) | .97 (.06) | .98 (.06) | .97 (.05) |
|  | D60 |  | .98 (.05) |  | 1.00 (.02) |  | .99 (.04) |  | .98 (.06) |  | .98 (.04) |  | .97 (.07) |
|  | D90 |  |  |  |  |  | .95 (.11) |  |  |  |  |  |  |

*Note*. Standard deviations are in parentheses.

Table S2. The mean RTs of the 360-degree search task merged across 15 YAs and 19 OAs.

|  |  | Longitude | | | | | | | | | | | |
| --- | --- | --- | --- | --- | --- | --- | --- | --- | --- | --- | --- | --- | --- |
|  |  | L150 | L120 | L90 | L60 | L30 | LR0 | R30 | R60 | R90 | R120 | R150 | LR180 |
| Latitude | U90 |  |  |  |  |  | 5.68 (0.62) |  |  |  |  |  |  |
|  | U60 |  | 5.23 (0.31) |  | 3.52 (0.24) |  | 3.42 (0.34) |  | 3.79 (0.31) |  | 5.34 (0.33) |  | 7.17 (0.68) |
|  | U30 | 5.59 (0.42) | 4.77 (0.33) | 3.95 (0.26) | 3.43 (0.21) | 1.54 (0.13) | 1.17 (0.08) | 1.50 (0.14) | 3.48 (0.21) | 4.14 (0.21) | 4.99 (0.31) | 6.31 (0.51) | 6.97 (0.58) |
|  | UD0 | 5.52 (0.43) | 4.56 (0.25) | 4.09 (0.24) | 4.05 (0.30) | 1.11 (0.08) | 1.37 (0.10) | 1.06 (0.06) | 4.05 (0.34) | 4.11 (0.26) | 4.58 (0.26) | 6.13 (0.56) | 6.96 (0.55) |
|  | D30 | 5.89 (0.46) | 4.80 (0.31) | 4.23 (0.23) | 3.66 (0.30) | 1.84 (0.18) | 1.24 (0.10) | 1.50 (0.16) | 3.77 (0.28) | 4.19 (0.26) | 5.04 (0.32) | 6.05 (0.46) | 6.85 (0.58) |
|  | D60 |  | 5.71 (0.30) |  | 4.46 (0.31) |  | 3.70 (0.27) |  | 3.84 (0.26) |  | 5.61 (0.34) |  | 7.53 (0.63) |
|  | D90 |  |  |  |  |  | 7.28 (0.67) |  |  |  |  |  |  |

*Note*. Standard errors are in parentheses.

Table S3. The mean correct response rate of the 360-degree search task for 15 YAs.

|  |  | Longitude | | | | | | | | | | | |
| --- | --- | --- | --- | --- | --- | --- | --- | --- | --- | --- | --- | --- | --- |
|  |  | L150 | L120 | L90 | L60 | L30 | LR0 | R30 | R60 | R90 | R120 | R150 | LR180 |
| Latitude | U90 |  |  |  |  |  | .98 (.04) |  |  |  |  |  |  |
|  | U60 |  | .98 (.06) |  | .98 (.04) |  | .99 (.03) |  | 1.00 (.00) |  | .99 (.03) |  | .98 (.05) |
|  | U30 | .99 (.03) | .99 (.03) | .97 (.06) | .98 (.07) | .97 (.10) | .99 (.03) | .99 (.03) | .98 (.10) | .99 (.03) | .98 (.04) | .99 (.03) | .99 (.03) |
|  | UD0 | .98 (.04) | .99 (.03) | .98 (.07) | .99 (.03) | .97 (.07) | .99 (.03) | .98 (.04) | .99 (.03) | .98 (.04) | .98 (.04) | .99 (.03) | .99 (.03) |
|  | D30 | .96 (.06) | .97 (.07) | .98 (.04) | .97 (.06) | .99 (.03) | .99 (.03) | .97 (.06) | .98 (.04) | .99 (.03) | .95 (.08) | .98 (.04) | .98 (.04) |
|  | D60 |  | .99 (.03) |  | 1.00 (.00) |  | .99 (.03) |  | .98 (.05) |  | .97 (.06) |  | .98 (.06) |
|  | D90 |  |  |  |  |  | .99 (.03) |  |  |  |  |  |  |

*Note*. Standard deviations are in parentheses.

Table S4. The mean correct response rate of the 360-degree search task for 19 OAs.

|  |  | Longitude | | | | | | | | | | | |
| --- | --- | --- | --- | --- | --- | --- | --- | --- | --- | --- | --- | --- | --- |
|  |  | L150 | L120 | L90 | L60 | L30 | LR0 | R30 | R60 | R90 | R120 | R150 | LR180 |
| Latitude | U90 |  |  |  |  |  | .93 (.11) |  |  |  |  |  |  |
|  | U60 |  | .99 (.04) |  | .99 (.04) |  | .99 (.04) |  | 1.00 (.00) |  | .97 (.05) |  | .99 (.03) |
|  | U30 | .95 (.09) | .99 (.04) | .98 (.05) | .98 (.05) | 1.00 (.00) | .98 (.05) | 1.00 (.00) | .99 (.04) | 1.00 (.00) | .99 (.03) | .95 (.10) | .97 (.07) |
|  | UD0 | .98 (.05) | .99 (.04) | 1.00 (.00) | .98 (.05) | .98 (.05) | .97 (.07) | .98 (.06) | .99 (.04) | .99 (.04) | .98 (.05) | .97 (.07) | .99 (.04) |
|  | D30 | .97 (.05) | .99 (.03) | .99 (.03) | .99 (.04) | .99 (.04) | .99 (.03) | .99 (.03) | .98 (.05) | .98 (.05) | .99 (.04) | .97 (.07) | .96 (.06) |
|  | D60 |  | .97 (.06) |  | .99 (.03) |  | .99 (.04) |  | .98 (.06) |  | .99 (.03) |  | .96 (.07) |
|  | D90 |  |  |  |  |  | .91 (.14) |  |  |  |  |  |  |

*Note*. Standard deviations are in parentheses.

Table S5. The mean RTs of the 360-degree search task for 15 YAs.

|  |  | Longitude | | | | | | | | | | | |
| --- | --- | --- | --- | --- | --- | --- | --- | --- | --- | --- | --- | --- | --- |
|  |  | L150 | L120 | L90 | L60 | L30 | LR0 | R30 | R60 | R90 | R120 | R150 | LR180 |
| Latitude | U90 |  |  |  |  |  | 4.73 (0.55) |  |  |  |  |  |  |
|  | U60 |  | 4.38 (0.41) |  | 3.37 (0.23) |  | 3.33 (0.37) |  | 3.90 (0.47) |  | 4.80 (0.49) |  | 5.31 (0.47) |
|  | U30 | 4.47 (0.38) | 3.93 (0.37) | 3.28 (0.33) | 3.10 (0.29) | 1.27 (0.14) | 0.93 (0.07) | 1.18 (0.18) | 3.62 (0.38) | 3.59 (0.29) | 4.01 (0.29) | 4.63 (0.37) | 4.81 (0.41) |
|  | UD0 | 4.25 (0.39) | 4.07 (0.39) | 3.88 (0.38) | 3.39 (0.26) | 1.07 (0.18) | 1.26 (0.22) | 1.01 (0.11) | 3.64 (0.37) | 3.87 (0.32) | 3.90 (0.31) | 4.10 (0.34) | 4.67 (0.33) |
|  | D30 | 4.62 (0.46) | 4.22 (0.36) | 3.77 (0.27) | 2.89 (0.31) | 1.36 (0.17) | 0.99 (0.13) | 1.22 (0.13) | 3.14 (0.32) | 3.90 (0.35) | 4.07 (0.38) | 4.52 (0.33) | 4.70 (0.42) |
|  | D60 |  | 5.04 (0.38) |  | 4.10 (0.46) |  | 3.03 (0.34) |  | 3.25 (0.26) |  | 5.10 (0.45) |  | 5.68 (0.49) |
|  | D90 |  |  |  |  |  | 6.41 (0.58) |  |  |  |  |  |  |

*Note*. Standard errors are in parentheses.

Table S6. The mean RTs of the 360-degree search task for 19 OAs.

|  |  | Longitude | | | | | | | | | | | |
| --- | --- | --- | --- | --- | --- | --- | --- | --- | --- | --- | --- | --- | --- |
|  |  | L150 | L120 | L90 | L60 | L30 | LR0 | R30 | R60 | R90 | R120 | R150 | LR180 |
| Latitude | U90 |  |  |  |  |  | 6.33 (0.98) |  |  |  |  |  |  |
|  | U60 |  | 5.93 (0.36) |  | 3.71 (0.30) |  | 3.45 (0.54) |  | 3.71 (0.42) |  | 5.73 (0.45) |  | 8.56 (1.09) |
|  | U30 | 6.46 (0.64) | 5.37 (0.44) | 4.51 (0.33) | 3.67 (0.30) | 1.73 (0.19) | 1.34 (0.11) | 1.73 (0.20) | 3.39 (0.23) | 4.60 (0.26) | 5.76 (0.44) | 7.54 (0.76) | 8.56 (0.83) |
|  | UD0 | 6.47 (0.65) | 4.92 (0.30) | 4.25 (0.32) | 4.60 (0.48) | 1.13 (0.07) | 1.44 (0.07) | 1.08 (0.06) | 4.36 (0.55) | 4.33 (0.39) | 5.08 (0.37) | 7.69 (0.82) | 8.75 (0.73) |
|  | D30 | 6.91 (0.68) | 5.30 (0.46) | 4.55 (0.34) | 4.34 (0.43) | 2.28 (0.27) | 1.42 (0.14) | 1.80 (0.26) | 4.21 (0.40) | 4.44 (0.38) | 5.84 (0.41) | 7.20 (0.68) | 8.46 (0.83) |
|  | D60 |  | 6.22 (0.42) |  | 4.82 (0.42) |  | 4.18 (0.38) |  | 4.29 (0.39) |  | 6.06 (0.48) |  | 8.86 (0.97) |
|  | D90 |  |  |  |  |  | 7.82 (1.13) |  |  |  |  |  |  |

*Note*. Standard errors are in parentheses.

Table S7. The mean normalized RTs of the 360-degree search task for 15 YAs.

|  |  | Longitude | | | | | | | | | | | |
| --- | --- | --- | --- | --- | --- | --- | --- | --- | --- | --- | --- | --- | --- |
|  |  | L150 | L120 | L90 | L60 | L30 | LR0 | R30 | R60 | R90 | R120 | R150 | LR180 |
| Latitude | U90 |  |  |  |  |  | .64 (.06) |  |  |  |  |  |  |
|  | U60 |  | .62 (.06) |  | .47 (.03) |  | .46 (.05) |  | .53 (.05) |  | .66 (.05) |  | .73 (.04) |
|  | U30 | .62 (.05) | .55 (.05) | .47 (.05) | .44 (.04) | .18 (.03) | .13 (.01) | .18 (.05) | .49 (.04) | .50 (.04) | .56 (.04) | .64 (.04) | .66 (.04) |
|  | UD0 | .59 (.04) | .57 (.05) | .54 (.05) | .48 (.03) | .15 (.02) | .18 (.03) | .15 (.02) | .52 (.06) | .54 (.04) | .54 (.03) | .57 (.04) | .65 (.04) |
|  | D30 | .64 (.05) | .59 (.04) | .54 (.04) | .41 (.04) | .20 (.03) | .15 (.03) | .18 (.03) | .44 (.04) | .53 (.03) | .56 (.05) | .63 (.04) | .64 (.04) |
|  | D60 |  | .70 (.04) |  | .57 (.05) |  | .43 (.04) |  | .46 (.04) |  | .69 (.04) |  | .76 (.04) |
|  | D90 |  |  |  |  |  | .86 (.04) |  |  |  |  |  |  |

*Note*. Standard errors are in parentheses.

Table S8. The mean normalized RTs of the 360-degree search task for 19 OAs.

|  |  | Longitude | | | | | | | | | | | |
| --- | --- | --- | --- | --- | --- | --- | --- | --- | --- | --- | --- | --- | --- |
|  |  | L150 | L120 | L90 | L60 | L30 | LR0 | R30 | R60 | R90 | R120 | R150 | LR180 |
| Latitude | U90 |  |  |  |  |  | .56 (.06) |  |  |  |  |  |  |
|  | U60 |  | .56 (.04) |  | .35 (.03) |  | .32 (.04) |  | .36 (.04) |  | .53 (.03) |  | .74 (.04) |
|  | U30 | .58 (.03) | .49 (.03) | .43 (.03) | .35 (.03) | .17 (.02) | .14 (.02) | .17 (.02) | .34 (.04) | .44 (.03) | .52 (.03) | .67 (.03) | .76 (.04) |
|  | UD0 | .58 (.03) | .46 (.03) | .40 (.03) | .44 (.05) | .11 (.01) | .15 (.02) | .11 (.01) | .41 (.05) | .41 (.04) | .47 (.03) | .67 (.03) | .79 (.04) |
|  | D30 | .63 (.04) | .48 (.03) | .43 (.04) | .40 (.04) | .21 (.02) | .14 (.01) | .17 (.03) | .40 (.04) | .43 (.04) | .55 (.04) | .65 (.04) | .75 (.04) |
|  | D60 |  | .57 (.03) |  | .44 (.04) |  | .39 (.04) |  | .39 (.03) |  | .56 (.04) |  | .78 (.04) |
|  | D90 |  |  |  |  |  | .66 (.04) |  |  |  |  |  |  |

*Note*. Standard errors are in parentheses.


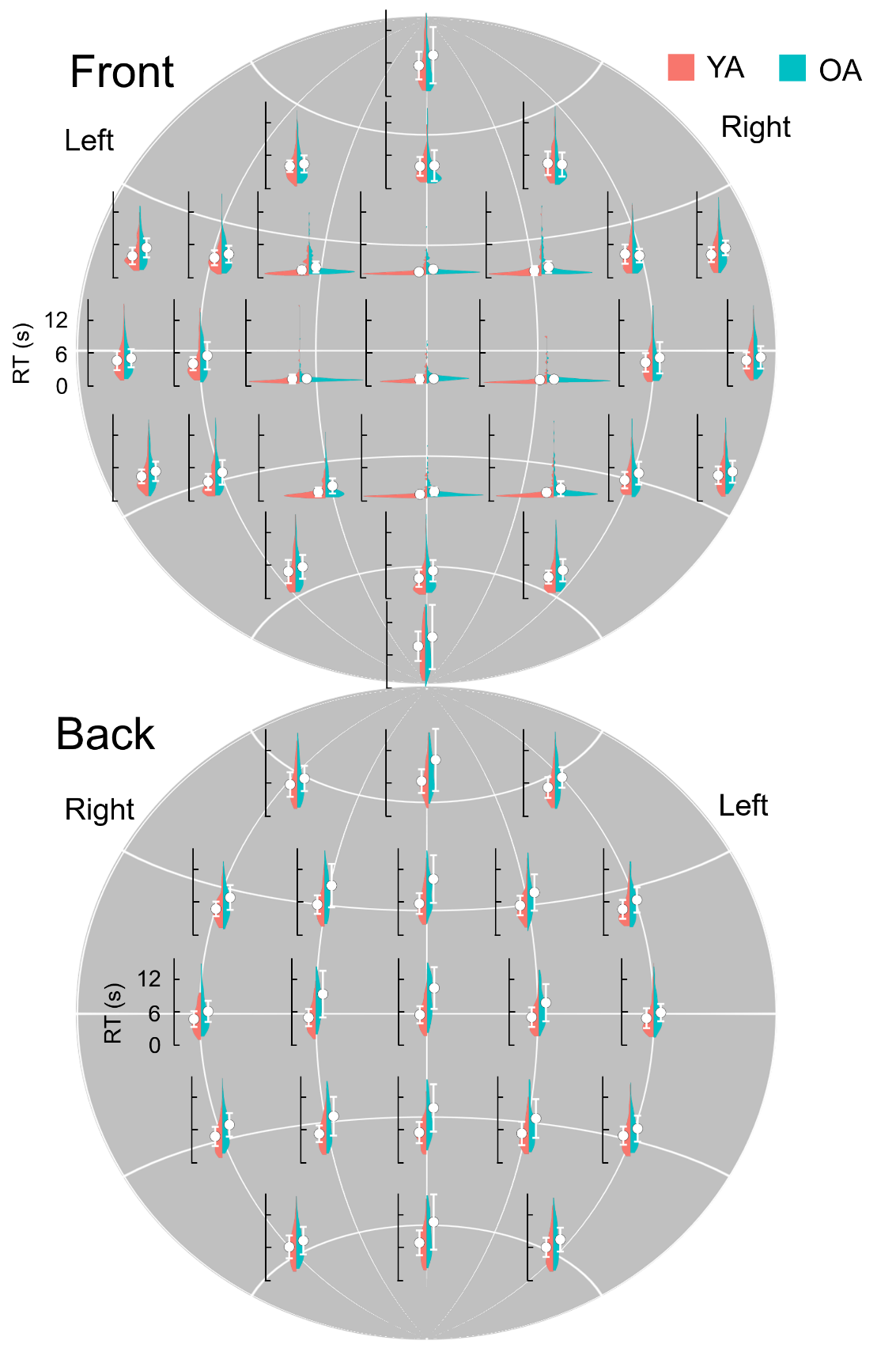


**Supplementary Figure S1. The means and standard deviations of the RTs in 15 YAs and 19 OAs.** White circles represent the mean RTs, and the error bars represent the standard deviations. Violin plots represent the Kernel density estimation.
